# Research progress on the hedgehog signalling pathway in regulating bone formation and homeostasis

**DOI:** 10.1111/cpr.13162

**Published:** 2021-12-16

**Authors:** Huan Zhou, Lei Zhang, Yue Chen, Chun‐Hui Zhu, Fa‐Ming Chen, Ang Li

**Affiliations:** ^1^ Key Laboratory of Shaanxi Province for Craniofacial Precision Medicine Research College of Stomatology Xi’an Jiaotong University Xi’an China; ^2^ Department of Periodontology College of Stomatology Xi’an Jiaotong University Xi’an China; ^3^ Department of Orthopaedic Surgery Xi'an Children's Hospital Xi’an China; ^4^ Department of Periodontology School of Stomatology State Key Laboratory of Military Stomatology National Clinical Research Center for Oral Diseases and Shaanxi Engineering Research Center for Dental Materials and Advanced Manufacture Fourth Military Medical University Xi’an China

**Keywords:** bone homeostasis, bone regeneration, hedgehog, mesenchymal stem cells, osteogenesis

## Abstract

Bone formation is a complex regeneration process that was regulated by many signalling pathways, such as Wnt, Notch, BMP and Hedgehog (Hh). All of these signalling have been demonstrated to participate in the bone repair process. In particular, one promising signalling pathway involved in bone formation and homeostasis is the Hh pathway. According to present knowledge, Hh signalling plays a vital role in the development of various tissues and organs in the embryo. In adults, the dysregulation of Hh signalling has been verified to be involved in bone‐related diseases in terms of osteoarthritis, osteoporosis and bone fracture; and during the repair processes, Hh signalling could be reactivated and further modulate bone formation. In this chapter, we summarize our current understanding on the function of Hh signalling in bone formation and homeostasis. Additionally, the current therapeutic strategies targeting this cascade to coordinate and mediate the osteogenesis process have been reviewed.

## Graphical abstract

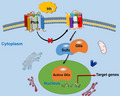

## 1 GENERAL ASPECTS OF HEDGEHOG SIGNALLING

Hedgehog (Hh) signalling pathway is a highly conserved pathway that is involved in embryonic development, tissue homeostasis and stem cell maintenance of invertebrates and vertebrates.^1^ The components of the Hh signalling pathway mainly include Hh ligand, patched receptor (Ptch), smoothened receptor (Smo), suppressor of fused (Sufu) and transcription factor glioma‐associated oncogene (Gli). In vertebrates, three Hh gene family members have been detected: sonic hedgehog (Shh), Indian hedgehog (Ihh) and desert hedgehog (Dhh).^2^ Ptch is a 12‐pass transmembrane receptor of Hh ligands, including two homologous genes, Ptch1 and Ptch2. Smo is a seven transmembrane protein that functions as a signal sensor. Generally, vertebrates contain three Glis proteins, namely, Gli1, Gli2 and Gli3, which are transcription factors with zinc finger structures. Usually, Gli1 and Gli2 function primarily as transcriptional activators, while Gli3 acts as a repressor of Hh signalling. Sufu is a negative regulator of the Hh pathway.

**FIGURE 1:**
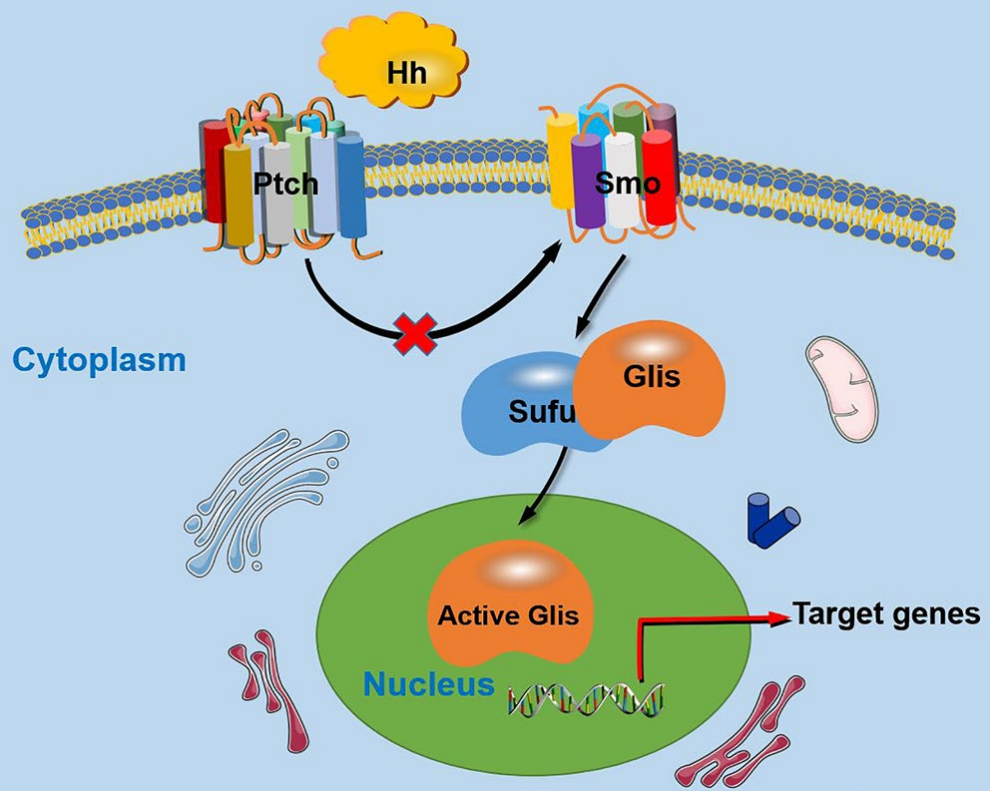
A simplified display of the Hh signalling pathway. In the presence of the Hh protein, the combination of Hh and Ptch abolishes the suppressive effects on Smo delivered by Ptch; then, Hh signalling is activated and the Hh signals are transducted into cells. After that, transcription factors Glis family are dissociated from a suppressive complex containing Sufu and further activated. Subsequently, the Hh signalling downstream target genes that contribute to certain cellular activities are modulated. Hh, hedgehog; Ptch, patched receptor; Smo, smoothened receptor; Gli, glioma‐associated oncogene; Sufu, suppressor of fused

Generally, the Hh pathway is triggered by binding of the Hh protein to its receptor Ptch. In the absence of the Hh ligands, Ptch is usually located around the primary cilia and suppresses the activity of Smo; when Hh protein bind to Ptch on the target cell, Ptch exits from the primary cilia; and then, this certain action relieves the suppressive effects on Smo delivered by Ptch, which results in the activation of Smo, thus Hh signalling is activated, further leading to the transduction of Hh signals into cells. Then, transcription factors in terms of the Glis family are activated, and Glis are dissociated from a suppressive complex containing Sufu. In the end, the Hh signalling downstream target genes that contribute to certain cellular activities are modulated (Figure 1).^3^ In addition, certain co‐receptors, like growth arrest specific (Gas), has been demonstrated to interact with Hh ligands to activate the Hh signalling, whereas for hedgehog interacting protein (HHIP), a membrane glycoprotein which could bind all the Hh ligands was found to negatively modulate the Hh signalling by preventing the interaction between Hh ligands and Ptch and finally attenuated the Hh signals.^4^

Several studies have indicated the indispensable functions of Hh signalling in bone formation and homeostasis, as their vital roles in modulating the osteogenesis of mesenchymal stem cells (MSCs), and key functions both individually as well as in coordination with other signalling cascades in terms of Wnt, BMP and parathyroid hormone‐related protein (PTHrP) during skeletal development and bone repair.^5^, ^6^, ^7^ More importantly, the dysregulation of Hh signalling could lead to bone‐related diseases in terms of osteoarthritis, osteoporosis and bone defects. In these settings, this review aims to organize and review the functions of Hh signalling in bone repair and regeneration.

## 2 REGULATION OF MSC OSTEOGENIC DIFFERENTIATION BY HH SIGNALLING

**FIGURE 2:**
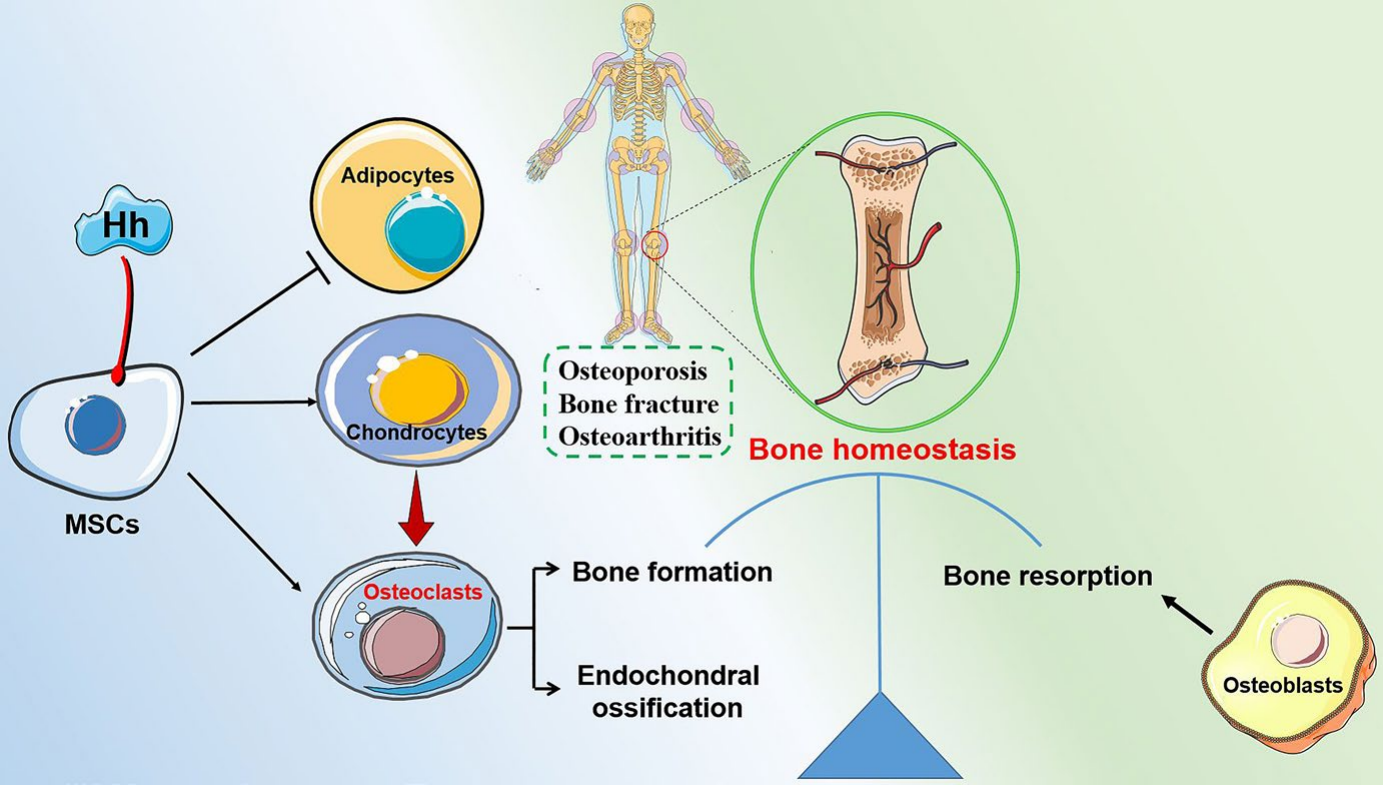
Hedgehog signalling plays an important role in regulating MSC differentiation and maintaining bone homeostasis. Hh signalling inhibits MSC differentiation into adipocytes, whereas it promotes their differentiation into chondrocytes and osteoblasts, further getting involved in maintaining bone homeostasis where osteoblasts mediate bone formation, and osteoclasts dominate the bone resorption. Additionally, the dysregulation of Hh signalling could lead to bone‐related diseases in terms of osteoarthritis, osteoporosis and bone fracture. MSC, mesenchymal stem cell

The osteogenic lineage commitment of MSCs is regulated by mechanical signals, paracrine factors, cytokines, chemokines and growth factors within their niche, which then activate a variety of signalling cascades, including Hh (Figure 2).^7^ A previously conducted study identified that recombinant N‐terminal Shh (ShhN) promoted the proliferation and osteogenic differentiation of rat bone marrow MSCs (BMMSCs) *in vitro*, as evidenced by enhanced ALP activity, increased osteogenesis‐related gene expression and matrix mineralization.^8^ Additionally, the implantation of MSCs overexpressing ShhN significantly accelerated bone formation *in vivo*; specifically, a 4‐mm segmental bone allograft model in immunodeficient mice was established, and the modified MSC administration significantly promoted bone defect reconstruction via improving donor cell survival and differentiation, along with scaffold revascularization at the bone defect site.^9^ Considering that Shh and Nel‐like 1 protein (Nell‐1) both possess osteoinductive potential, a combination therapy using ShhN with Nell‐1 was established, and this particular combination strategy was demonstrated to markedly facilitate the osteogenic differentiation of adipose‐derived MSCs (hASCs) when compared with either cytokine alone. Additionally, the pro‐osteogenic function delivered by Nell‐1 alone could be abolished in response to the Hh signalling inhibition with a Smo antagonist (cyclopamine). This particular combination cytokine strategy may be of potential therapeutic benefit for bone regeneration.^10^ Additionally, Hh signalling was found to be involved in the osteoblastic differentiation deficiency of BMMSCs under high glucose (HG) conditions. In specific, HG delivered an inhibitory effect on BMMSC osteogenic differentiation; however, the addition of recombinant Shh alleviated the inhibitory function induced by HG, where cells transfected with Shh lentivirus demonstrated increased matrix mineralization nodules, higher ALP activity and expression levels of bone sialoprotein(BSP), osteopontin (OPN) and bone morphogenetic protein 4 (BMP‐4). Additionally, a tooth extraction model in diabetes mellitus rats was established to verify the *in vitro* results. As expected, Shh administration promoted bone formation within the extraction socket.^11^, ^12^ In a recent study, Hh signalling was reported to be involved in the osteogenesis process, and the Hh gene was required for the loading‐mediated osteogenic differentiation of the murine MSC line C3H10T1/2.^13^

Dexamethasone, a well‐known promoter for osteoblast differentiation, was found to enhance ALP activity and type I collagen expression and up‐regulate Shh expression levels during the osteoblast differentiation process. Interestingly, both the mRNA and protein expression of Ihh and Gli1 were down‐regulated.^14^ The Hh pathway also participated in the BMMSC osteogenic differentiation process induced by simvastatin. Specifically, simvastatin administration resulted in an enhanced osteogenic differentiation capacity, indicated by up‐regulated expression of COL1, ALP and osteocalcin (OCN), and increased ALP activity. More importantly, BMMSCs treated with simvastatin expressed higher levels of Ihh and Gli1, and more nuclear translocation of Gli1 was observed. Contrasting effects were observed after the BMMSCs were exposed to cyclopamine (an Hh signalling inhibitor), indicating that simvastatin promotes BMMSC osteogenic differentiation, at least in part, via the Hh pathway.^15^ During mandibular development, ciliary protein Ift88 was found to participate in chondrogenesis and osteogenesis at least partially via the Shh pathway.^16^ Additionally, low‐level laser irradiation was also found to promote osteoblast proliferation via Hh signalling.^17^ Whereas oxidative stress was demonstrated to inhibit MSC osteogenic differentiation (in part) by modulating Hh signalling, where H_2_O_2_ prevented the Shh‐induced osteogenic differentiation of MSCs (murine primary MSCs and other MSC lines), as demonstrated by decreased expression of ALP, Osterix (OSX) and BSP.^18^ Recently, a subpopulation of MSCs named Gli1^+^ cells was found to promote type H vasculature (CD31^hi^EMCN^hi^ type vessels) formation to accelerate bone defect healing, which means significance to tissue homeostasis and regenerative repair, and further studies demonstrated that the underlying mechanisms may lie in that Gli1^+^ cells promoted angiogenesis via the Gli‐HIF‐1a signalling.^19^

Additionally, a variety of microRNAs (miRNAs) have been reported to get involved in the osteogenesis process mediated by Hh signalling. For example, miR‐342‐3p was identified as a therapeutic agent that can accelerate the osteogenic differentiation of human umbilical mesenchymal stem cells (UCMSCs) by down‐regulating Sufu to activate Shh signalling.^20^ Another study also verified that miR‐342‐3p was highly expressed in hUCMSCs during osteogenic differentiation, and miR‐342‐3p overexpression markedly up‐regulated the expression levels of osteogenic‐related genes (ALP, Cbfa1 and OPG) by activating Hh signalling.^21^ miR‐196a could reverse the MSC osteogenic differentiation obtained in osteoporosis mice by targeting GNAS to further activate Hh signalling,^22^ whereas miR‐467g was found to be an inhibitor for osteoblast differentiation, and could negatively regulate the osteogenesis process via Ihh/runt‐related transcription factor 2(RUNX2) signalling.^23^

## 3 HH SIGNALLING AND BONE‐RELATED DISEASE

The Hh pathway plays a crucial role in skeletal development and bone repair, and the dysregulation of Hh signalling could lead to bone‐related diseases, including osteoarthritis, osteoporosis and bone fracture. For example, osteoporosis results from decreased bone formation by osteoblasts in parallel with increased bone resorption by osteoclasts (Figure 2). Under this context, modulating Hh signalling to manipulate osteoprogenitor cells to augment the osteogenic differentiation potential and enhance bone formation properties is of great significance.

### 3.1 Osteoporosis

Osteoporosis is a metabolic bone disease represented by continuous destruction of bone mass and microstructure due to the imbalance of bone formation and resorption. Based on the current understanding, dysfunction of MSCs with impaired osteogenic potential contributes to osteogenesis disorders, especially the osteoporosis development. For example, oxidative stress was verified to deliver its suppressive effect on MSC osteogenic differentiation and facilitate age‐related osteoporosis by inhibiting Hh signalling.^18^ A previous study reported that miR‐196a was poorly expressed when its direct downstream target GNAS was overexpressed in osteoporosis mice. After transfection with miR‐196a mimic, the MSCs (isolated from osteoporosis mice) displayed significantly elevated ALP vitality, increased bone formation ability and higher expression levels of osteogenesis‐related factors, including ALP, RUNX2 and OPN. More importantly, Smo expression was significantly up‐regulated, while the expression of Ptch and GNAS was markedly down‐regulated. Collectively, miR‐196a promoted the osteogenic differentiation of MSCs by down‐regulating GNAS to activate the Hh pathway.^22^ In addition, laminin α2 (LAMA2) inhibition was also demonstrated to enhance MSC osteogenic differentiation and inhibit their adipogenic differentiation by regulating the Hh pathway, implying that regulation of Hh signalling might be a potential strategy for osteoporosis treatment.^24^ Also, Ihh‐Ptch1 signalling was verified to have an important function in postnatal bone homeostasis because Ptch1‐deficient (Ptch+/‐) cells demonstrated augmented osteoblast differentiation, as verified by up‐regulated expression of RUNX2, indicating that Ptch1 may represent a promising modulatory target for osteoporosis treatment.^25^

### 3.2 Osteoarthritis

The dysregulation of Hh signalling can lead to osteoarthritis, which is characterized by progressive degeneration of articular cartilage, and in Ihh‐depleted mice, the expression of osteoarthritis‐related markers, including MMP‐13 and collagen type X, was significantly down‐regulated. Thus, the inhibition of Ihh signalling could be regarded as a promising strategy to prevent or treat osteoarthritis.^26^ Additionally, a study conducted by Ruiz‐Heiland G concluded that Hh signalling blockade could be protective in that this particular inhibition treatment could block the formation of collagen type X and hypertrophic chondrocytes and inhibit osteophyte formation.^27^ Recently, a Smo‐specific inhibitor named taladegib was verified to be a promising agent for osteoarthritis treatment, since it controlled chondrocyte hypertrophy by down‐regulating the expression of MMP13, collagen type X and RUNX2 via Smo/Gli1 signalling.^28^ Woods S et al. found that the expression of miR‐324‐5p was increased in osteoarthritic cartilage. Further study verified that miR‐324‐5p regulated Hh signalling is conserved in humans and mice, yet the specific regulatory mechanism is distinct, where miR‐324‐5p modulated osteogenesis in human MSCs by targeting Gli1 and Smo and further regulated Hh signalling, whereas in mouse C3H10T1/2 cells, miR‐324‐5p regulated Hh signalling by directly targeting Gpc1 but not Smo or Gli1.^29^ Naproxen (Npx), a nonsteroidal anti‐inflammatory drug (NSAID) used for osteoarthritis treatment, was verified to affect the osteogenic differentiation of human MSCs via Hh signalling. Npx had a dual role in promoting MSC hypertrophic differentiation while inhibiting their osteogenic differentiation; this discovery of the underlying mechanisms of Npx and other NSAIDs possesses far‐reaching significance for improving the clinical therapeutic effect of osteomyelitis treatment.^30^

### 3.3 Bone fracture

A previous study indicated that inhibition of Ca2�/calmodulin (CaM)‐dependent protein kinase kinase 2 (CaMKK2) could accelerate fracture healing by stimulating Ihh signalling; more specifically, treatment with STO‐609 (an inhibitor of CaMKK2) accelerated endochondral ossification in the central callus, and the expression levels of Ptch1 and Gli1 were markedly elevated by 6.5‐fold and 2.5‐fold, respectively.^31^ Cigarette smoke extract (CSE) has been reported to induce the integrity loss of primary cilia, which specialize in Hh signalling, thus inhibiting MSC osteogenic differentiation. However, resveratrol treatment rescued the inhibitory effects induced by CSE and promoted MSC osteogenic differentiation *in vitro* by affecting Hh target gene expression, which suggests promising therapeutic alternatives for fracture treatment in smokers, especially for delayed fracture healing.^32^ Additionally, Hh signalling was observed to be involved in impaired bone healing in the setting of diabetes mellitus. A previous study found that inhibition of Hh signalling suppressed the expansion of injury‐induced mouse skeletal stem cells (mSSCs), further impairing bone healing in diabetic mice. Then, a slow release hydrogel was utilized to precisely deliver recombinant Ihh/Shh to the local fracture site, which led to an accelerated fracture repair effect because the impaired expansion and osteogenic potential of mSSCs in response to injury were restored.^33^ A previously conducted study demonstrated that Shh‐positive and Gli1‐positive cells were localized along the surface of the newly formed bone; further study identified that Shh and Gli1 were co‐localized with RUNX2 and OSX at the fracture site, implying that Shh‐Gli1 signalling regulates intramembranous and endochondral ossification processes within bone fracture healing.^34^

## 4 SMALL MOLECULES/BIOLOGICAL MATERIALS IN HEDGEHOG SIGNALLING AND OSTEOGENESIS REGULATION

In recent years, much progress has been made in discovering and applying small molecules or bioactive materials to regulate stem cell commitment, and target‐based manipulation provides substantial insights into therapeutic strategies for committing MSCs to tissue regeneration.^35^ In this context, employing small molecule agents/bioactive materials to modulate Hh signals represents a promising therapeutic approach for the treatment of bone‐related diseases, and these natural or synthetic agents are promising for promoting osteogenesis for bone repair/regeneration (Table 1).

**TABLE 1:** Small molecules or biological materials involved in hedgehog signalling and osteogenesis regulation

Small molecules	Effect	Experimental model	Comments	Ref.
Simvastatin	Activation	Rat BMMSCs	Increased COL1, ALP and OCN; Up‐regulated Gli1 and Ihh	^15^
STO−609	Enhanced	Femurs fractures of male C57BL6/J mice	Increased bone mineralization; Elevated Ihh, Gli1, and Ptch1	^31^
Resveratrol	Activation	Human MSCs	Enhanced RUNX2, BMP−2, OPG and RANKL; Up‐regulated Gli2,	^32^
Taladegib	Suppression	Chondrocyte hypertrophy	Inhibited type X collagen, MMP−13 and RUNX2; Up‐regulated Smo and Gli1	^28^
Naproxen	Activation	Human MSCs	Decreased ALP and COL1A1; Up‐regulated COL10A1 and OPN; Increased Ihh, Ptch1, Gli1 and Gli2	^30^
Purmorphamine	Activation	Human endometrial stem cells on collagen/hydroxyapatite scaffold	Increased COL1, RUNX2 and ALP; Up‐regulated Gli1 and Ptch	36, 37
20s	Activation	Mouse MSCs	Increased BSP, BMP−2, Col1A2 and RUNX2; Up‐regulated Gli1, Ptch and Shh	^42^
SS	Activation	PDLSCs	Increased OCN, OSX and RUNX2; Up‐regulated Smo and Gli1	^43^
Oxy133	Activation	Rabbit BMMSCs; Critical‐sized cranial defects in rabbits	Increased ALP, RUNX2, COL and OSX; Promoted bone regeneration in bone defects	^44^
Oxy49	Activation	Rabbit BMMSCs; Critical‐sized cranial defects in rabbits	Increased COL1, OSX, OCN, OPN and ALP; accelerated bone regeneration in bone defects	^47^
SAG	Activation	NMCCs; Critical‐size mouse calvarial defect	Increased BSP, OCN and VEGF, Increased bone volume, bone thickness, and blood vessel number as well as density	^50^ ^51^
Hh‐Ag 1.7	Activation	MSCs (C3H10T1/2 cells)	Increased OPN, OCN, IBSP and ALP; Up‐regulated osterix/Sp7 and Gli1	^55^
GDC−0449	Suppression	Cyclic loading‐induced ulnar stress fracture model	Decreased bone volume and mineral density, fracture callus blood vessel density; Decreased IBSP and ALP; Down‐regulated Shh, Gli1, Ptch1 and HHIP	^56^
Astragaloside IV	Activation	Human osteoblast‐like cells	Enhanced cell proliferation and migration; Up‐regulated Shh and Gli1	^57^
Resveratrol	Activation	Human MSC line (SCP−1)	Enhanced AP activity, matrix mineralization; RUNX2, BMP−2, OPG and RANKL; Up‐regulated Gli2 and restored cilia integrity	^32^
BGC	Activation	Rat BMMSCs	Increased BMP−2, OCN, RUNX2 and ALP; Up‐regulated Smo and Gli1	^58^
MNTs	Activation	Human MG63 osteoblasts	Enhanced BMP−2, ALP and RUNX2; Up‐regulated Shh, Smo and Gli	^59^
PCL	Activation	Dental pulp stem cells	Enhanced BMP−2, BMP−4, FOXA2 and Ptch1	^60^
nHA	Activation	MC3T3‐E1 lineage; Mouse pre‐osteoblastic cells,	Lead to low profile of RANKL transcripts; Up‐modulated Shh and Smo, down‐regulated Ptch	^61^

Note

20s, 20(S)‐hydroxycholesterol; SS, 22(S)‐hydroxycholesterol combined with 20(S)‐ hydroxycholesterol; PDLSCs, periodontal ligament stem cells; SAG, smoothened agonist; NMCCs, primary neonatal mouse calvarial cells; BGC, bioactive glass‐ceramic; MNTs, micro‐/nanotextured topographies; PCL, fluorapatite‐modified polycaprolactone nanofiber; nHA, nano‐scaled hydroxyapatite‐blasted titanium.

### 4.1 Purmorphamine

Purmorphamine, a Smo receptor agonist, was demonstrated to induce the osteogenic differentiation of human endometrial stem cells seeded on a collagen/HA scaffold, where the ALP level and RUNX2 expression were both up‐regulated.^36^ Besides, the pro‐osteogenic effect of purmorphamine was demonstrated, to an extent, similar to that delivered by BMP‐4. Purmorphamine administration increased cellular proliferation and up‐regulated osteogenic gene expression by activating Hh signalling, resulting in bone formation.^37^ Currently, therapeutic strategies utilizing purmorphamine for the treatment of bone‐related disease have been widely studied due to its bone regenerative properties.^38^, ^39^

### 4.2 Oxysterols

Oxysterols are natural molecules, as oxidized cholesterol derivatives, oxysterols bind to Smo and then activate Hh signalling to modulate the osteogenic process.^40^, ^41^ Among them, the most potent is 20(S)‐hydroxycholesterol (20S), which was found to promote the osteogenic differentiation, whereas it inhibits the adipocyte differentiation of MSCs isolated from compact bones of broiler chickens (cBMSCs). After adding the Hh inhibitor cyclopamine, the pro‐osteogenic and anti‐adipogenic effects induced by 20S were completely reversed, indicating that 20S plays a vital role in the cBMSC differentiation by regulating Hh signalling.^42^ Additionally, a previous study conducted by Lee JS investigated the bone regenerative potential of combined oxysterols (SS, 22(S)‐hydroxycholesterol combined with 20(S)‐hydroxycholesterol) and found that the combined oxysterol SS accelerated the osteogenic differentiation of periodontal ligament stem cells (PDLSCs) *in vitro*, as represented by augmented ALP activity and osteogenesis‐related markers. In an *in vivo* study, SS implantation remarkably promoted bone healing in a tooth extraction bone defect model. Regarding the underlying mechanisms, the researchers found that SS treatment resulted in up‐regulated expression of nuclear receptors for oxysterols (LXRs, liver X receptor α and β), as well as their target genes (ATP‐binding cassette transporter A1, ABCA1). Additionally, the expression of Hh signalling proteins, including Smo and Gli1, was up‐regulated.^43^ More importantly, a reciprocal reaction between LXRs and Hh signalling was confirmed, and si‐LXRα and si‐LXRβ treatment attenuated the protein levels of Smo and Gli1. In turn, the inhibition of Hh signalling also down‐regulated the expression levels of LXRα and LXRβ. These studies collectively suggest that a combination of oxysterols may represent a promising strategy for bone regeneration.^43^ Additionally, Oxy133 and Oxy49, two analogues of naturally occurring oxysterols, were demonstrated to promote MSC osteogenic differentiation *in vitro* and accelerate bone regeneration *in vivo* to a certain degree, with an efficacy comparable to that mediated by BMP‐2.^44^, ^45^, ^46^, ^47^ Recently, researchers designed a nanoparticulate agonist consisting of palmitic acid and oxysterol, which was designed to bind Smo to activate Hh signalling, and this may be a prospective strategy for the treatment of bone defects.^48^

### 4.3 Smoothened agonist (SAG)

Smoothened agonist has been identified as an activator of Hh signalling, which facilitated the translocation of Smo from the cytoplasm to the primary cilium and stabilized it in its active form.^49^ Lee and colleagues found that SAG administration led to accelerated osteoblast differentiation *in vitro* and promoted calvarial bone healing *in vivo*, which may be applied for bone defect treatment.^50^ Further studies found that the administration of SAG combined with Nell‐1 significantly accelerated calvarial bone defect healing, as demonstrated by increased bone volume and bone thickness as well as increased defect vascularization.^51^ A previous study found that Kruppel‐like factor 4 (KLF4) inhibited osteoblast differentiation by repressing basal Hh activity. After SAG treatment, the decreased expression of osteoblastic genes and mineralization delivered by KLF‐4 was significantly up‐regulated.^52^ Also, the administration of SAG combined with helioxanthin derivative markedly promoted bone formation and finally achieved bone healing (in a rat femur bone defect model) without cell transplantation.^53^ Under this context, the combination therapy involved with SAG and other molecules, like BMP‐2, may represent a promising strategy for bone repair.^54^

### 4.4 Other small molecules

Hh‐Ag 1.7, a non‐peptidyl small molecule agonist of Hh signalling (binding to Smo), was found to increase Gli1 expression, and promote the osteoblast differentiation of C3H10T1/2 cells. Further studies demonstrated that Hh‐Ag 1.7 functioned synergistically with BMP‐2 to enhance cell osteoblast differentiation. Considering these impressive outcomes, Hh‐Ag 1.7 may be an attractive choice for application in bone healing settings.^55^ A previous study revealed that the Hh pathway was involved in osteogenesis and angiogenesis in the settings of stress fracture healing, where Hh signalling was activated in response to stress fracture; however, after that the Hh antagonist GDC‐0449 (vismodegib, acts directly on Smo) was utilized, the mineral apposition rate, bone formation rate and fracture callus blood vessel density were markedly decreased. Additionally, the expression of key Hh signalling key genes, including Shh, Gli1 and Patch1, was significantly down‐regulated.^56^ Astragaloside IV is an effective agent isolated form Astragali Radix (Chinese medicine). A previous study found that astragaloside IV promoted the proliferation and migration of osteoblasts to facilitate osseointegration by Hh pathway.^57^ Also, resveratrol treatment could improve the osteogenic differentiation potential of MSCs *in vitro* via alternating the expressions of Hh target genes, which provides promising therapeutic alternatives for the treatment of bone diseases.^32^

### 4.5 Biological materials

Bioactive glass‐ceramic (BGC), a classical bone tissue engineering scaffold, was found to accelerate the proliferation and osteogenic differentiation of BMMSCs. Further study identified that Hh/Smo/Gli1 signalling was involved in BGC‐mediated osteogenesis, and the expression of Smo and Gli1 was significantly up‐regulated in the BGC group compared with the control group. After treatment with cyclopamine, the expression of osteogenesis‐related genes and Hh signalling members was markedly down‐regulated.^58^ In a previous study, MG63 cells seeded onto micro‐/nanotextured topographies (MNTs) decorated with TiO2 nanotubes exhibited markedly enhanced cell adhesion, proliferation and osteogenic differentiation, and Hh–Gli1 signalling was found to play key roles in cell biological responsiveness to MNTs.^59^ Fluorapatite (FA)‐modified polycaprolactone (PCL) nanofiber is an odontogenic/osteogenic inductive tissue engineering scaffold, which was reported to positively regulate the osteogenic differentiation of dental pulp stem cells (DPSCs) partially via the Hh pathway.^60^ Nano‐scaled hydroxyapatite‐blasted titanium (nHA) was also utilized for osteoblast differentiation due to its anti‐inflammatory potential, and Shh signalling was verified to be involved in the osteoblast process mediated by nHA, which could be regulated to guarantee osteoblast activity towards osteogenesis.^61^

## 5 HH SIGNALLING INTERACTS WITH OTHER SIGNALLING PATHWAYS TO REGULATE BONE FORMATION

**FIGURE 3:**
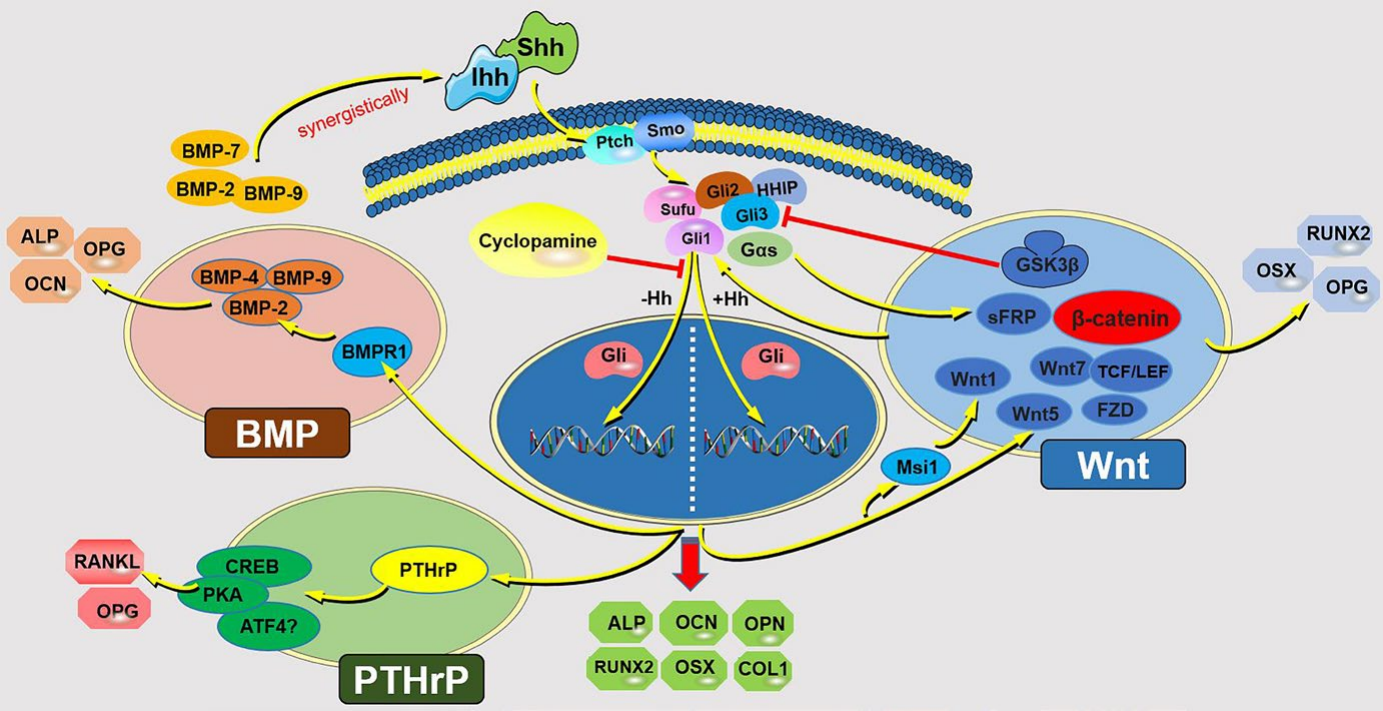
Hedgehog signalling interacts with other signalling pathways (Wnt, BMP, PTHrP) to regulate bone formation. Hh and Wnt signalling can functionally antagonistic or work synergistically through common regulators in terms of sFRP‐1, HHIP, Gαs, Msi1 Gli3, Wnt5, GSK3 and β‐catenin during osteogenesis. Hh and BMP signalling usually has a synergistic effect on the osteogenic process in that they can induce each other's expression or enhance each other's function (Shh, Ihh, BMP‐2, BMP‐7 and BMP‐9). Ihh‐PTHrP axis is critical to bone homeostasis, not only via the endochondral pathway but also in the bone remodelling process, which could modulate the expression of OPG and RANKL. PTHrP, parathyroid hormone‐related protein; sFRP‐1, secreted frizzled‐related protein 1; BMP, bone morphogenetic protein; HHIP, hedgehog interacting protein; Gas growth arrest specific; Msi1, Musashi 1; GSK3, glycogen synthase kinase‐3; OPN, osteopontin; RANKL, receptor activator of nuclear factor‐κ B ligand

A variety of signalling cascades are involved in osteogenesis‐related cell fate decisions, and osteogenesis is well orchestrated by various signalling pathways such as Wnt, BMP PTHrP and Hh (Figure 3). In this setting, gaining a better understanding of the interaction of the “osteogenic signalling network” for tissue engineering is of great significance, and the distinct window for each signalling in terms of timing and the threshold level of its activation is pivotal.^62^

### 5.1 Hh‐Wnt axis

Regarding the interaction of Hh and Wnt signalling, a variety of studies have reported that Hh and Wnt signalling is functionally antagonistic through common regulators, such as secreted frizzled‐related protein 1(sFRP‐1).^63^, ^64^, ^65^ Hh‐Wnt pathways were involved in the Nell‐1‐induced osteogenic differentiation of human adipose‐derived stem cells (ADSCs), where key factors of Hh signalling (Ihh, Gli1, Gli2 and Smo) and sFRP‐1 (an antagonist of Wnt) were down‐regulated during the osteogenesis process, whereas antagonists of Hh signalling including Gli3 and HHIP were up‐regulated.^66^ Gli3, the transcriptional repressor of Hh signalling, was verified to be a direct downstream target gene of Wnt/β‐catenin signalling, and activation of Wnt pathway could up‐regulate Gli3, further suppressing Hh signalling.^67^ Gαs has been validated to be the downstream of Smo and upstream of Gli transcription factors, and it could suppress Hh signalling. In Gαs‐/‐embryos, Hh target gene expression was higher, whereas Wnt target gene expression was lower. Thus, modulating Gαs could maintain a balance between Hh and Wnt‐β‐catenin signalling.^68^ Additionally, a previous study validated that Hh signalling negatively interacted with Wnt signalling during the osteogenic differentiation of human umbilical cord blood (UCB)‐derived MSCs (hUCB‐MSCs). Where Hh signalling functioned as a negative regulator of osteogenic differentiation of hUCB‐MSCs via regulating RNA‐binding Musashi (Msi1), which acted as a downstream modulator of Hh signalling. In addition, Msi1 down‐regulated the expressions of Wnt1 and miR‐148 family, further leading to decreased osteogenic potential.^69^ Mak KK et al. demonstrated that Ihh and Wnt signalling interacted with each other during osteoblast differentiation, and β‐catenin is the required downstream of Ihh signalling for OSX expression, which is essential for osteoblast differentiation.^70^

However, Hu H et al. found that Wnt signalling functioned as a downstream signal of the Ihh pathway in the development of osteoblast lineage, and Hh‐induced osteogenesis required activated Wnt signalling,^71^ where Hh and Wnt signalling orchestrate the osteoblast process in a sequential manner, and initially, Hh signal initiated the expression of RUNX2 and Col1a1. Then, Hh activated the Wnt signalling which is required for OSX expression and osteoblast differentiation. In summary, Hh and Wnt signals collectively modulate osteoblast development in an orchestrated way. In addition, oxysterols were verified to exert a stimulatory effect on the osteogenic differentiation of embryonic stem cells (ESCs) by Hh signalling, which triggered mitochondrial activity and further activated Wnt/β‐catenin signalling. Collectively, these two pathways collaborated in the promotion of ESC osteogenesis in response to oxysterols.^72^ Magnesium was found to promote distraction osteogenesis by targeting Ptch protein to activate Hh signalling. Further RNA sequencing studies demonstrated that the Hh pathway was the upstream signalling of the alternative Wnt pathway. Thus, Hh‐alternative Wnt signalling co‐worked in the distraction osteogenesis process and could be regulated to enhance bone formation.^73^ GRK2 was identified as an essential regulator of skeletogenesis, and mutations in GRK2 could lead to skeletal ciliopathies by impairing both Hh signalling and Wnt signalling.^74^ Tang et al. examined the effect of lithium chloride (LiCl) on the differentiation of BMMSCs and identified that LiCl markedly accelerated cell osteogenic differentiation and inhibited adipogenic differentiation simultaneously. Further studies identified that these processes were modulated by the Hh pathway synergistically with Wnt signalling.^75^ More importantly, Hh and Wnt signalling could intersect intracellularly via common regulators in terms of GSK3 and Sufu.^76^, ^77^ Although much progress has been made for the interaction between Hh and Wnt signalling, the interaction between Hh and Wnt signalling remains complex, and to be uncovered, these contradictory results might be related to cell types, signal strength, signal timing and conditions.^78^ Whether Hh signalling facilitates or inhibits the Wnt pathway or vice versa during the osteogenesis process remains controversial and needs further exploration.

### 5.2 Hh‐BMP axis

In addition to Wnt signalling, other pathways also contribute to Hh‐induced bone formation. Of note, BMP signalling has been validated to be required or has a positive impact on Hh‐mediated osteogenesis.^79^ It was reported that BMP‐dependent Hh signalling was required for calvarial bone defect repair, and it regulated the interplay between suture MSCs and osteoclasts, which are both crucial for calvarial bone homeostasis and injury repair.^80^ In addition, Ihh and BMP‐2 were verified to deliver a synergistically effect on the osteogenic differentiation of human MSCs.^81^ Additionally, Hh signalling was demonstrated to play a regulatory role in the BMP‐9‐induced osteogenic differentiation of MSCs, for cyclopamine (an Hh signalling inhibitor) treatment significantly decreased the expression of osteogenesis‐related markers, including ALP, OCN, OPN, and the BMP‐9‐induced transcriptional activity of Smad1/5/8, whereas the expression levels of these molecules were remarkably up‐regulated by purmorphamine (an Hh signalling agonist).^82^ Generally, it has been validated that BMP and Shh signalling have a synergistic effect on MSC osteogenic differentiation in that they can induce each other's expression or enhance each other's function in the osteogenesis process.^83^ However, Jiang Q et al. reported that Shh signalling and BMP signalling have antagonistic effects on the osteogenic differentiation of stem cells from apical papilla (SCAPs), where the activation of Shh signalling by recombinant Shh‐N protein or by overexpression of Smo inhibited the osteo/dentinogenic differentiation of SCAPs. Further study demonstrated that Shh signalling was repressed by BMP signalling; more importantly, the decreased osteo/dentinogenic differentiation of SCAPs (mediated by Shh signalling) was enhanced.^84^ These contradictory results regarding the interaction and function of Shh and BMP signalling in MSC differentiation might be related to cell types and conditions, which remain to be explored with more in‐depth and detailed research.

### 5.3 Ihh–PTHrP axis

Previous studies have demonstrated that the Ihh‐PTHrP feedback pathway was critical to the endochondral ossification process.^85^ Additionally, Ihh and PTHrP could work together to commit MSCs towards the osteoblastic lineage by inducing RUNX2.^86^ Mak KK et al. found that the activity of Hh signalling was down‐regulated progressively as osteoblasts matured in the postnatal bone, and up‐regulating the Hh axis selectively in mature osteoblasts resulted in increased bone formation and excessive bone resorption, further leading to osteopenia. However, down‐regulating the Hh signalling (in mature osteoblasts) led to increased bone mass and, more importantly, reduced bone loss. Further molecular studies verified that Hh signalling indirectly modulated osteoclast bone formation and resorption processes by up‐regulating osteoblast expression of PTHrP, which in turn regulated receptor activator of nuclear factor‐κ B ligand (RANKL) expression, further getting involved in maintaining bone homeostasis where osteoblasts mediate bone formation, osteoclasts dominate the bone resorption.^87^ In addition, Ihh and PTHrP signals were both sensitive to static pressure, and the interaction between Ihh and PTHrP axis was found to be involved in the chondrogenic and osteogenic differentiation of condylar chondrocytes within the pressure microenvironment.^88^ Collectively, the Ihh‐PTHrP interaction loop is of significance for bone homeostasis, not only via the endochondral pathway but also in the bone remodelling process. Thus, regulating MSC osteoblastic differentiation and modulating the Ihh‐PTHrP axis may be of potential future therapeutic benefit.

Additionally, other signalling pathways was reported to be involved in osteogenesis mediated by Hh signalling. A previous study demonstrated that simvastatin promoted the osteogenic differentiation of BMMSCs partially by Hh signalling, while researchers found that simvastatin could enhance Gli1 activity even in the presence of the Hh signalling inhibitor cyclopamine, indicating that other potential pathways may be involved in simvastatin‐induced osteogenesis. Then, the potential proteins interacting with Gli1 were explored using mass spectrometric analysis, and Hh signalling was found to interact with other pathways in terms of MAPK, Hippo, insulin or glucagon signalling by regulating the expression of the related molecules Ppp2r1a, Rac1, Etf1 and XPO1/CRM1.^15^

## 6 CONCLUSIONS AND FUTURE PROSPECTS

Based on the above‐mentioned studies, Hh signalling functioned significantly in bone homeostasis, which is involved in osteoblast differentiation and osteogenesis, and the dysregulation of Hh signalling may lead to bone‐related diseases in terms of osteoarthritis, osteoporosis and bone defects. Under this context, certain pharmacologic agents that designed to regulate Hh signalling are already available for disease treatment or in development to safeguard skeletal health. For example, after regulating the Hh signalling precisely, the osteoarthritis degeneration pace would be slower; bone regeneration within defect could be enhanced.^89^ Additionally, a variety of small molecules or biological materials have been utilized to modulate Hh signalling to facilitate osteogenesis. However, the utilization of Hh morphogens or Smo agonists is facing multiple challenges in terms of high‐dose requirements, low specificity and stability, short‐acting time and potential side effects *in vivo*. Under this context, it is necessary to develop alternative efficacious strategies to modulate Hh signalling utilizing nano‐carriers towards to faster and safer bone repair/regeneration. For example, extracellular vesicles (EVs), as cell‐secreted lipid bilayer structures, can be manipulated as therapeutic tools for any molecule of interest including Shh, to promote osteogenesis.^90^ As naturally occurring secreted vesicles, EVs possess certain advantages over other carrying agents (like liposomes), including low propensity to trigger immune rejection, no toxicity concern and high stability.^91^ Additionally, attention needs to be paid to fundamental problems of fine‐tuning the duration and strength of the Hh axis at appropriate timing, together with other signalling cascades in terms of the Wnt, BMP and PTHrP axes, because the regulatory mechanisms involved in the Hh signalling are complicated and cell type‐specific. Additionally, novel mechanisms are continuously being identified; and each new finding usually triggers another question.^92^ These challenges need to be addressed before the potential of these approaches can be fully realized to facilitate bone formation and maintain bone homeostasis^93^.

## CONFLICT OF INTEREST

The authors declare that they have no competing interests.

## AUTHOR CONTRIBUTION

HZ, ZL and YC conceptualized the study; HZ, ZL and CH‐Z wrote original draft; F‐MC and AL contributed to writing—review and editing. All authors read and approved the final manuscript.

## Data Availability

Data sharing is not applicable to this article, as no new data were created or analysed in this paper.
